# Effect of Zr Addition on the Mechanical Properties and Superplasticity of a Forged SP700 Titanium Alloy

**DOI:** 10.3390/ma14040906

**Published:** 2021-02-14

**Authors:** Dong Han, Yongqing Zhao, Weidong Zeng

**Affiliations:** 1 School of Materials, Northwestern Polytechnical University, Xi’an 710072, China; zengwd@nwpu.edu.cn; 2 Northwest Institute for Nonferrous Metal Research, Xi’an 710016, China

**Keywords:** SP700 alloy, Zr, microstructure, tensile properties, superplasticity

## Abstract

The present study focuses on the effect of 1% Zr addition on the microstructure, tensile properties and superplasticity of a forged SP700 alloy. The results demonstrated that Zr has a significant effect on inhibiting the microstructural segregation and increasing the volume fraction of β-phase in the forged SP700 alloy. After annealing at 820 °C for 1 h and aging at 500 °C for 6 h, the SP700 alloy with 1% Zr showed a completely globular and fine microstructure. The yield strength, ultimate tensile strength and tensile elongation of the alloy with optimized microstructure were 1185 MPa, 1296 MPa and 10%, respectively. The superplastic deformation was performed at 750 °C with an elongation of 1248%. The improvement of tensile properties and superplasticity of the forged SP700 alloy by Zr addition was mainly attributed to the uniform and fine globular microstructures.

## 1. Introduction

The developed SP700 alloy, a β-rich α-β titanium (Ti) alloy, demonstrates high mechanical properties, pronounced superplasticity and excellent corrosion resistance, which make it an ideal structural material for applications in aerospace industries [1,2,3]. The wrought SP700 alloy generally has an extremely fine α+β duplex microstructure and is designed to improve the mechanical properties and the workability of the commercial Ti-6Al-4V alloy [4,5]. The duplex microstructures of SP700 alloy or other α + β Ti alloys are commonly optimized by the methods of heat treatment (annealing and aging) and/or adding of specific alloy elements [3,6,7,8,9]. One reason is that the microstructures typically rely on the allotropic transformation from a high-temperature bcc (β) phase to a lower temperature hcp (α) phase through the heating process [6]. With a reasonable annealing and aging process, the primary lamellar microstructure in the α + β phase region changes into an equiaxed or globular one due to static recrystallization [10]. The other reason is that the specific element can stabilize a lesser or greater volume fraction of β-phases. The addition of stable β-phase elements can decrease the transformation temperature of β-phase and lower the superplastic temperature. Combining the above two typical methods, we can obtain an optimized microstructural morphology and an adjusted volume fraction ratio of α-phase and β-phase.

The nominal composition of SP700 alloy is Ti-4.5%Al-3%V-2%Fe-2%Mo (wt.%) [3,4,11,12,13]. Fe and Mo are chosen as the stable elements of β-phase, while Al and V act as the stable roles of α-phase [6,12,13]. The β-phase transformation in SP700 alloy is generally performed at 900 °C, and the superplastic behavior is commonly achieved around 800 °C [11]. It has been reported that the SP700 alloy with fine microstructure (grain size of 0.5~3 μm) shows superplastic elongation of 796% at 775 °C [4]. However, the SP700 alloy still faces the problem of further improvement of mechanical properties and superplasticity. The mechanical properties of SP700 alloy are strongly dependent on the existing states (size, volume fraction and distribution) of α and β phases [4,14]. Zr, as a neutral element, can make the chemical composition uniform, eliminate the eutectic region and improve the mechanical properties [15]. For example, based on first-principle calculations, α Ti-Zr alloy has a similar stability over a wide range of temperature [16]. However, it has been reported that Zr is a stabilizing β-phase element, which is prone to solute in β-phase and promote the growth of β-phase [17]. In addition, Zr can decrease the β-phase transformation temperature, and a lower temperature of β-phase transformation is helpful in decreasing the superplastic temperature [18]. As a result, the manufacturing efficiency can be reasonably improved and the processing cost can be reduced by Zr addition.

In the present work, we added Zr with a low melting point and low cost into SP700 alloy. The effects of Zr addition on the microstructure, mechanical properties and superplasticity of the forged SP700 alloy were analyzed and studied. The purpose of this work is to improve the mechanical properties and superplasticity of the SP700 alloy to satisfy the high requirements of aerospace applications for complex structural materials.

## 2. Materials and Experimental Procedure

The original materials for the investigation were forged SP700 and SP700Zr alloys. Chemical compositions of the two alloys are listed in Table 1.

The casting and forging processes were completed by the BAOTI Group Co. Ltd. (BaoTi company, Baoji, China). The ingot casting underwent two rounds of vacuum consumable melting. The size of the original ingot casting was φ 600 mm × 50 mm. After this, three heats of open forging were carried out on 2 × 10^7^ N fast forging equipment. The deformation mode was three upsetting and three drawing. The deformation temperatures were 1100, 1030 and 930 °C, respectively. The forging was completed in the two-phase region, with a deformation mode of two upsetting and two drawing and a deformation temperature of 870–875 °C. The thickness of blank size after forming was 160 mm.

Heat treatment of the forged SP700 and SP700Zr alloys was carried in a vacuum tube furnace by annealing at a higher temperature for complete β-phase transformation and then aging at a lower temperature to get a better distribution of α-phase. The heat treatment schedules were designed according to the references listed in Table 2.

Microstructures of the alloy were observed on an optical microscope (OM, Olympus GX71, Olympus company, Tokyo, Japan). The sample for OM observation was mechanically ground on SiC paper and subsequently polished to a mirror-like surface. The polished sample was then etched in a mixture solution of 5%HF + 10%HNO_3_ + 85%H_2_O. Tensile tests were conducted on an Instron 5869 testing machine (Instron company, Boston, MA, USA) and performed at different temperatures (25, 730, 750, 770 and 790 °C) and strain rates (1 × 10^−2^, 5 × 10^−3^, 1 × 10^−3^, 5 × 10^−4^ and 1 × 10^−4^ s^−1^). The tensile sample was designed as a dog-bone shape with a gauge size of a diameter of Ø 5 mm and length of 25 mm; the gauge length was machined with the direction parallel to the length of the forged plate. The morphologies of the fracture surfaces after superplastic deformation were observed by the transmission electron microscope (TEM, JEM-F200, JEOL, Tokyo, Japan). Samples for TEM observation were subjected to mechanical grinding with SiC paper to about 50 μm and polished into a mirror-like surface; they were then prepared by twinjet electro-polishing at a temperature below 20 °C. The polishing solution was a mixture of 6% perchloric acid, 60% methyl alcohol and 34% n-butyl alcohol.

## 3. Results and Discussion

### 3.1. Microstructures

**Figure 1:**
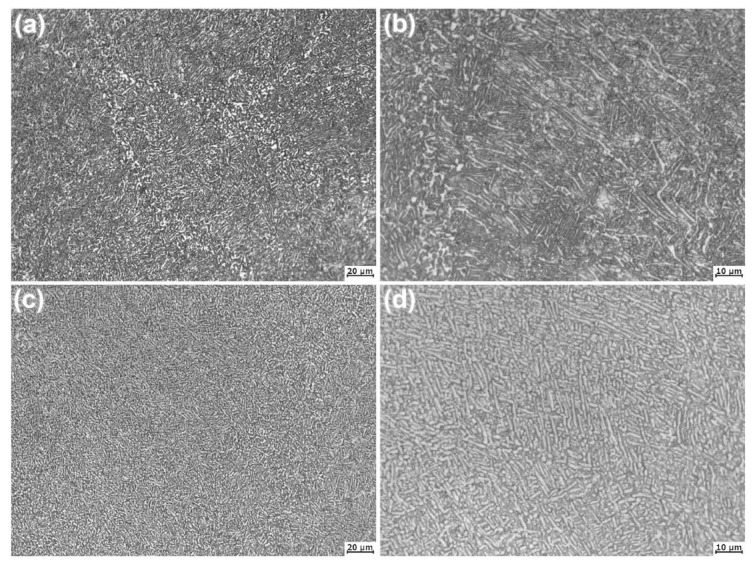
Optical microstructures of the (**a**) SP700 and (**c**) SP700Zr alloys; (**b**,**d**) are the locally enlarged microstructures of (**a,c**), respectively.

Figure 1 shows the optical microstructures of the forged SP700 and SP700Zr alloys. Both the forged alloys consist of two contrast phases. The two phases are in strip shapes and arranged at intervals with each other. The α-phases are black in color, while the β-phases are white. In the forged SP700 alloy, as shown in Figure 1a,b, besides the uniform interval of the arranged α and β eutectoid structures, the coarse and/or spherical primary α-phases are nonuniformly distributed in the microstructure. In contrast, the microstructure of the forged SP700Zr alloy, as shown in Figure 1c,d, is more uniform; the volume fraction of the β-phase is increased and the length of the two phases becomes smaller than that in the forged SP700 alloy. This reveals that Zr refines the α and β phases and increases the number of β-phases in forged SP700 alloy. The microstructural changes mainly arise from the fact that Zr decreases the temperature of β-phase transformation [18]; as a result, during the forging process under high temperature, more β phase can exist after forging in the SP700Zr alloy.

**Figure 2:**
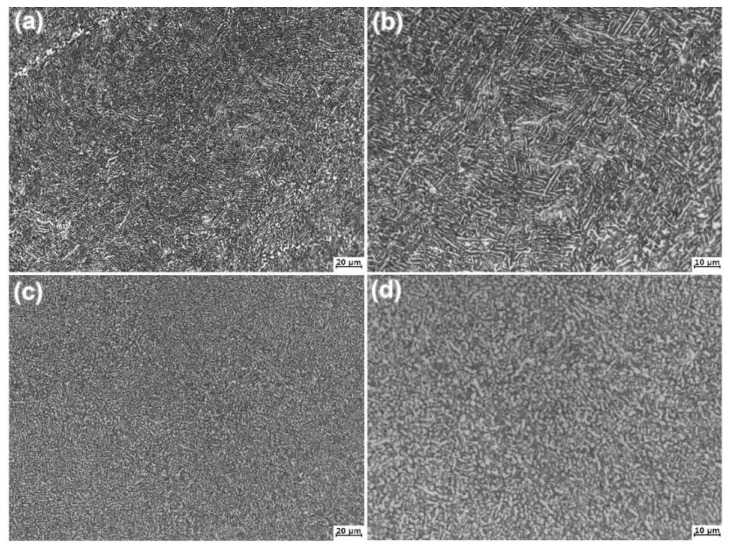
Optical microstructures of the (**a**) SP700 and (**c**) SP700Zr alloys after HT1 heat treatment; (**b**,**d**) are the locally enlarged microstructures of (**a,c**), respectively.

Figure 2 shows the optical microstructures of the forged SP700 and SP700Zr alloys annealing at 710 °C for 1 h (HT1). The nonuniform microstructure in SP700 alloy becomes unobvious, and the volume fraction of coarse primary α-phases is decreased. Meanwhile, the long lamellar α and β phases gradually change into short ones because of the static recrystallization. The static recrystallization phenomenon is also observed in SP700Zr alloy. However, the static recrystallization process is incomplete in the two alloys.

**Figure 3:**
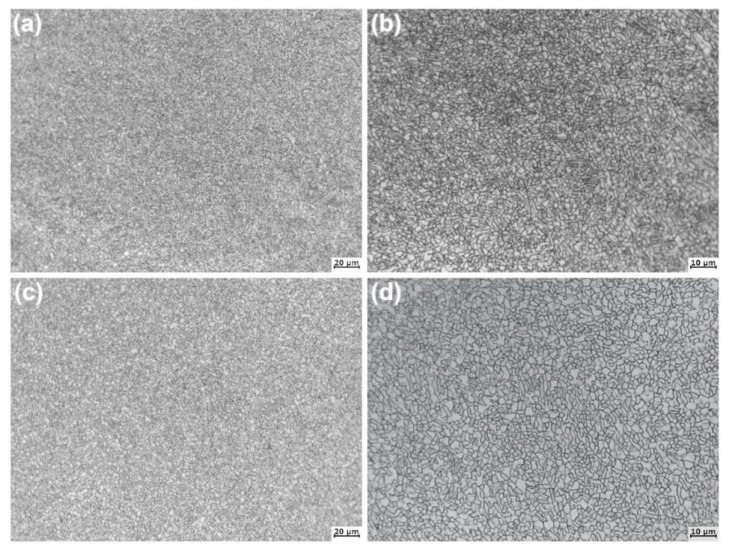
Optical microstructures of the (**a**) SP700 and (**c**) SP700Zr alloys after HT2 heat treatment; (**b**,**d**) are the locally enlarged microstructures of (**a,c**), respectively.

Figure 3 shows the optical microstructures of the forged SP700 and SP700Zr alloys annealing at 820 °C for 1h (HT2). After annealing at 820 °C, the primary α-phase volume fraction decreases, and a morphologic change of equiaxed microstructure is observed. This means that the strip α-phases and β-phases convert into short strip and/or spherical shapes, and the strip α-phases are diminished while the spherical β-phases are gradually increased. In Figure 3c,d, almost all the α-phases have been transformed into β-phases in forged SP700 (Figure 3a,b) and SP700Zr alloys. In other words, a completely static recrystallization microstructure can be performed in both alloys. In addition, the β-phase structure in forged SP700Zr alloy is more equiaxed and uniform, even though the structure is a little coarser than that in forged SP700 alloy when comparing Figure 3a with Figure 3c. In addition to the above differences, the grain size in both alloys is around 2 μm, which is far smaller than the requirement of 10 μm for superplasticity.

**Figure 4:**
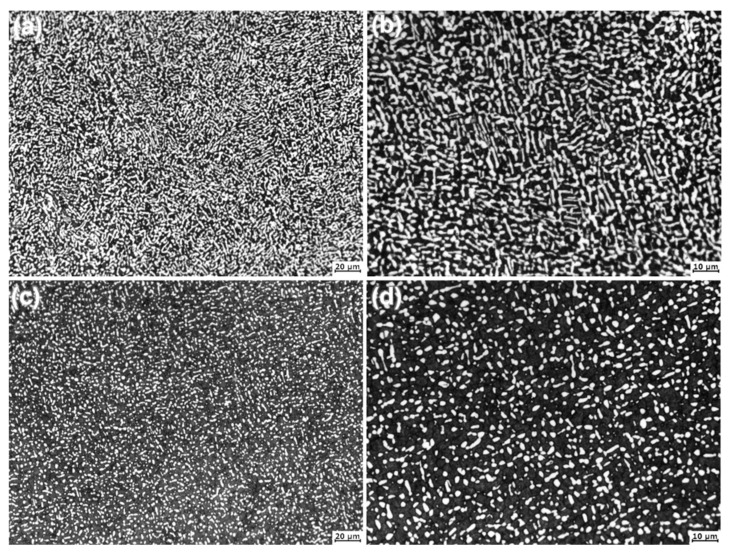
Optical microstructures of the (**a**) SP700 and (**c**) SP700Zr alloys after HT3 heat treatment; (**b**,**d**) are the locally enlarged microstructures of (**a**,**c**), respectively.

After annealing and aging heat treatment (HT3), the forged alloys present well uniform duplex microstructures, as shown in Figure 4. The α-phases precipitated and distributed dispersedly in the matrix (primarily consisting of β-phases). In SP700 alloy, most of the precipitated α-phases are in strip shapes, while most with smaller size are spheroidized in the SP700Zr alloy and distributed around the boundaries of β-phases. Accordingly, the SP700Zr displays a much more obviously equiaxed and globular microstructure. The larger number and grain size of β-phase as well as less α-phase in SP700Zr alloy indicate that Zr should be a β-phase stabilized element.

### 3.2. Mechanical and Superplastic Properties

Table 3 summarizes the values of yield strength, ultimate tensile strength and elongation of the forged SP700 and SP700Zr alloys in different heat treatment conditions at room temperature and a strain rate of 1 × 10^−3^ s^−1^. After HT1 and HT2 heat treatments, the SP700 alloy shows better strength and elongation than the SP700Zr alloy. However, after HT3 heat treatment, the SP700Zr alloy shows superior tensile strength and unchangeable elongation as compared with the SP700 alloy. In addition, the alloys in the HT3 condition possess the highest tensile strengths but have a lower elongation than those in the HT1 and HT2 conditions. This reveals that a proper heat treatment is an effective method to improve the tensile properties of SP700 alloy. This is mainly attributed to the redistribution of β-phases and α-phases by heat treatment. The higher strength of SP700 alloy in the HT1 condition, while becoming smaller in the HT3 condition compared with the strength of SP700Zr alloy, indicates the β-phases are much softer than the α-phases. The higher strength of SP700Zr alloy should be attributed to the fine grain size of α-phases and the solid solution strengthening effects from infinite solute Zr in Ti alloy. In addition, more homogeneous and uniform deformation can take place over the entire polycrystalline material with a uniform and globular structure. As a result, Zr increases the strength without decreasing the ductility. Table 4 summarizes the values of yield strength, ultimate tensile strength and elongation of the forged SP700 and SP700Zr alloys in HT3 conditions at elevated temperatures and a strain rate of 1 × 10^−3^ s^−1^.

**Figure 5:**
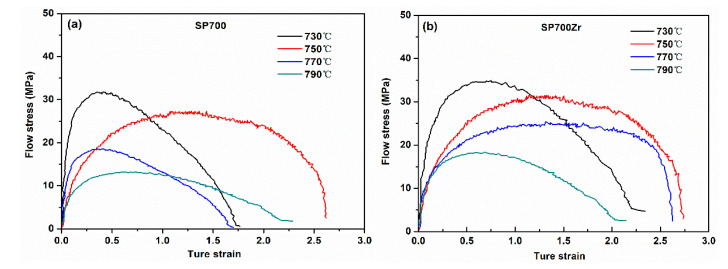
Tensile stress-strain curves at elevated temperatures of (**a**) SP700 and (**b**) SP700Zr alloys.

The flow stress-strain curves at different elevated temperatures (730~790 °C) and strain rates (1 × 10^−2^–1 × 10^−4^ s^−1^) were tested. Here we provide the flow stress-strain curves of the SP700 and SP700Zr alloys in the HT3 condition at elevated temperatures (730~790 °C) and a typical strain rate of 1 × 10^−3^ s^−1^ (Figure 5a,b). By increasing the temperature, the flow stress is gradually decreased, while the tensile strain increases initially and then decreases. Superplasticity is achieved at 750 °C in both alloys, which is indicated from the stress-strain curves. Furthermore, the SP700Zr alloy presents a superior superplasticity due to its larger tensile strain than that of the SP700 alloy. This reveals that Zr is a favorable element to improve the superplasticity of the SP700 alloy.

**Figure 6:**
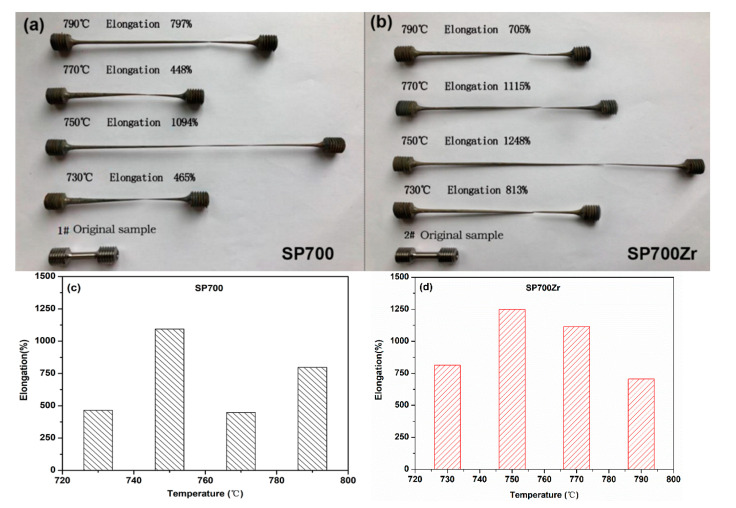
Photographic images and tensile elongations of the (**a**,**c**) SP700 and (**b**,**d**) SP700Zr samples before and after tensile tests at different elevated temperatures.

Figure 6a,b respectively show the photographic images of the tensile samples of SP700 alloy and SP700 Zr alloy in HT3 conditions before and after fracture at different temperatures (from 730 to 790 °C). Figure 6c,d summarize the tensile elongation at different temperatures of SP700 alloy and SP700Zr alloy in HT3 conditions. It is clear that superplastic deformation occurs at 750 °C. However, the tensile elongation of the SP700Zr alloy, 1248%, is much higher than that of the SP700 alloy, whose elongation is 1094%. Compared with previously reported results (796% at 775 °C) [4], the superplastic elongation of our work is far larger and the temperature is 45 °C lower. It is known that the formation of a fine globular structure with the grain size d < 10 μm in α + β titanium alloys usually results in their superplasticity at temperatures above 800 °C (T > 0.5 Tm) and strain rates below 10^−3^ s^−1^ [2,19,20,21]. Furthermore, the refinement structure with the grain size d < 1 μm leads to a shift in the superplasticity temperature or strain rate to lower temperatures and/or higher strain rates [22,23]. In the present study, for SP700 and SP700Zr alloys, a superplasticity elongation larger than 1000% can be achieved at a temperature of 750 °C, and such temperature is far lower than most superplastic Ti alloys [4].

**Figure 7:**
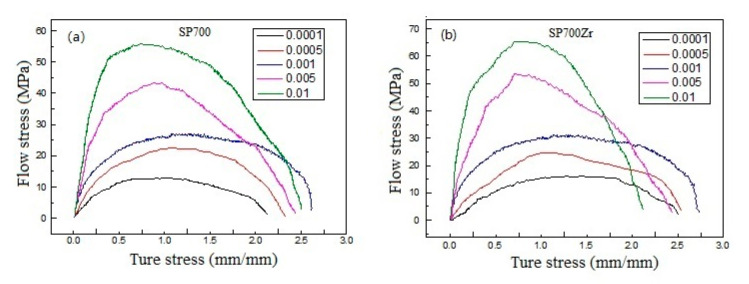
Tensile stress-strain curves at different strain rates of (**a**) SP700 and (**b**) SP700Zr alloys.

**Figure 8:**
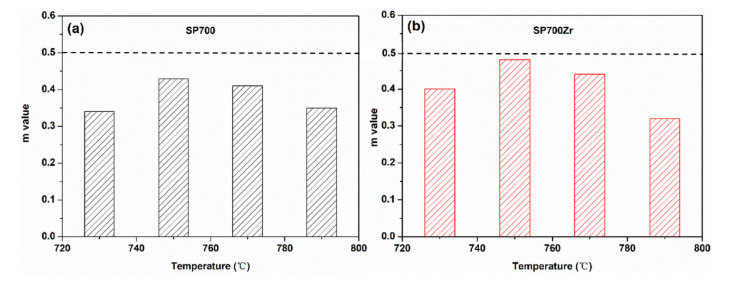
Strain rate sensitivity (m) values of the (**a**) SP700 and (**b**) SP700Zr alloy at different elevated temperatures.

The strain rate sensitivity value, m, which is measured from the analysis of stress-strain rate curves, is a function of the logarithmic values of the stress and strain rate [24]:

(1) $$m={\frac{\partial ln\sigma }{\partial ln\dot{\varepsilon }}|}_{\varepsilon ,T}$$

where *σ* and $\dot{\varepsilon }$ are the stress and strain rate for a given value of strain *ε* (0.5) and temperature T. Tensile stress-strain curves at different strain rates (including strain rates of 0.0001, 0.0005, 0.001, 0.005, 0.01) for the SP700 and SP700Zr alloys are displayed in Figure 7a,b. Based on the tensile stress-strain curves at different strain rates and temperatures, the derived m values for the SP700 and SP700Zr alloys are displayed in Figure 8a,b, respectively. It can be seen that the optimal m values are found at a temperature of 750 °C in both alloys, which is consistent with that of the largest elongation in the two alloys. Compared with the SP700 alloy (m value is 0.43), the SP700Zr alloy has a much larger m value of 0.48, which is more approximate to 0.5. For polycrystalline metallic materials, the m value is an important parameter to predict the superplastic deformation mechanism [25,26]. When the m value reaches 0.5, grain boundary sliding (GBS) should be the dominant deformation mechanism during the plastic deformation of metals and alloys [25]. Therefore, the large m value of 0.48 in the SP700Zr alloy indicates that the GBS is the dominate deformation mechanism controlling the superplasticity of the SP700Zr alloy.

**Figure 9:**
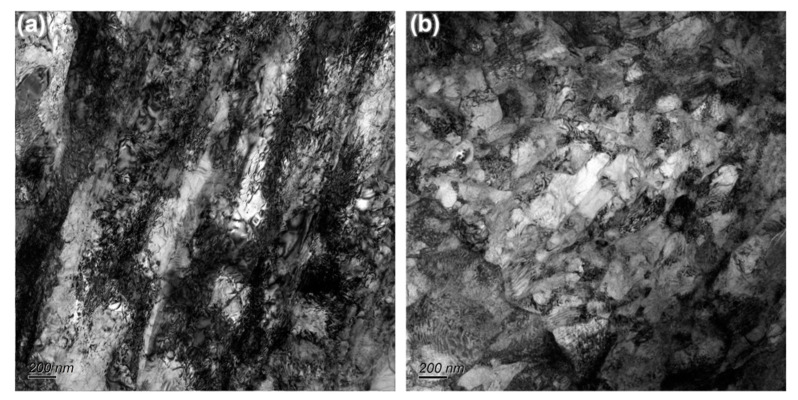
TEM images of microstructure of the forged (**a**) SP700 and (**b**) SP700Zr alloys in HT3 conditions after tensile fracture at 750 °C and 1 × 10^−3^ s^−1^.

Figure 9a,b display the TEM images of the forged SP700 and SP700Zr alloys in HT3 conditions after superplastic fracture, respectively. It is clear that in addition to the GBS behavior, many more dislocations in the SP700 alloy are observed than that in the SP700Zr alloy. The aggregation of dislocations results in the concentrated stress and strain, leading to the origin or nucleation of the microcracks or microvoids, which are easily linked with each other to grow into larger cracks; thus, plastic instability and earlier fracture of the alloys can occur [27]. It has been proven that dislocation activity more often occurs within the α-phase [28]. The intragranular deformation, i.e., dislocation creep, existing at α-phases is shown to accommodate the β-grain boundary sliding aided by the cavities and voids [26]. As shown in the SP700 alloy (Figure 9a), the high density of strip-like dislocations in α-phases and the non-equiaxed microstructure is believed to be harmful to a sustainable GBS, thus leading a lower superplasticity. However, in the SP700Zr alloy, fewer dislocations are observed in α-phases because of the limited dislocation storage of the fine α-phase. Furthermore, near-equiaxed fine grains with uniform distribution exhibit good deformation compatibility, thus minimizing void formation and coalescence during superplastic deformation and leading to a larger elongation to failure [6]. The uniform equiaxed β grains are helpful to accommodate the strain concentration of β-phase boundaries and are responsible for the sustainable GBS. Therefore, the uniform structure with globular/equiaxed β-phase and fine α-phase is the main reason that the SP700Zr alloy achieves a better superplasticity.

## 4. Conclusions

(1) Zr could inhibit the microstructural segregation, which provided the forged SP700 alloy with a much more uniform and globular microstructure.

(2) After annealing at 820 °C for 1 h and aging at 500 °C for 6 h, Zr stabilized the β-phase and enhanced the formation of equiaxed β-phase and refined α-phase in forged SP700 alloy.

(3) Zr improved the yield strength and ultimate tensile strength of SP700 alloy from 1107 ± 4.5 MPa and 1203 ± 3.5 MPa to 1185 ± 6.5 MPa and 1296 ± 5.0 MPa, respectively, without decreasing the tensile elongation (around 10%).

(4) After Zr addition, the superplastic elongation of the SP700 alloy was increased from 1094 ± 19% to 1248 ± 23% at 750 °C. This was attributed to the uniform equiaxed/globular fine microstructure, which are responsible for the GBS.

## Figures and Tables

**Table 1:** Chemical composition of SP700 and SP700Zr alloys.

Alloys	Actual Chemical Compositions (wt.%)					
	Ti	Al	V	Mo	Fe	Zr
SP700	Balance	4.5	3.0	2.0	2.0	0
SP700Zr	Balance	4.0	3.2	2.0	2.0	1.0

**Table 2:** Heat treatment schedules for the SP700 and SP700Zr alloys.

No.	Heat Treatment Schedules
HT1 (higher temperature 1)	710 °C 1 h Air Cooling (AC)
HT2 (higher temperature 2)	820 °C 1 h AC
HT3 (higher temperature 3)	820 °C 1 h + 500 °C 6 h AC

**Table 3:** Mechanical properties of SP700 and SP700Zr at RT.

Alloys	Heat Treatment	Mechanical Properties		
		Ultimate Tensile Strength (MPa)	Tensile Yield Strength (MPa)	Elongation (%)
SP700	HT1	1002 ± 2.5	965 ± 1.6	16 ± 0.3
	HT2	969 ± 6.2	904 ± 7.5	17 ± 0.3
	HT3	1203 ± 3.5	1107 ± 4.5	10 ± 0.5
SP700Zr	HT1	962 ± 4.5	926 ± 6.0	17 ± 0.5
	HT2	964 ± 6.0	861 ± 7.2	15 ± 0.5
	HT3	1296 ± 5.0	1185 ± 6.5	10 ± 0.3

**Table 4:** Mechanical properties of SP700 and SP700Zr in HT3 conditions at elevated temperatures.

Alloys	Temperature (°C)	Mechanical Properties		
		Ultimate Tensile Strength (MPa)	Tensile Yield Strength (MPa)	Elongation (%)
SP700	730	31 ± 0.6	28 ± 0.5	465 ± 0.5
	750	27 ± 0.3	24 ± 0.5	1094 ± 0.6
	770	18 ± 0.5	16 ± 0.6	448 ± 0.5
	790	13 ± 0.1	11 ± 08	797 ± 1.0
SP700Zr	730	34 ± 0.8	30 ± 0.6	813 ± 0.8
	750	31 ± 0.3	28 ± 0.2	1248 ± 0.5
	770	25 ± 0.1	22 ± 0.6	1115 ± 0.7
	790	18 ± 0.3	16 ± 0.2	705 ± 0.8

## Data Availability

The data used to support the findings of this study are available from the corresponding author upon request.
